# Prospective Comparative Study of Topical Tacrolimus and Sirolimus for the Treatment of Pigmentary Keratitis in Pug Dogs

**DOI:** 10.3390/vetsci13010047

**Published:** 2026-01-05

**Authors:** Diana Sarmiento Quintana, Inmaculada Morales Fariña, Jéssica González Pérez, Manuel Morales Doreste, José Raduan Jaber, Juan Alberto Corbera

**Affiliations:** 1 Hospital Clínico Veterinario, Facultad de Veterinaria, Universidad de Las Palmas de Gran Canaria, 35413 Las Palmas, Spain; 2 Dioftalmo, Oftalmología Veterinaria, 35003 Las Palmas, Spain; 3 Instituto Universitario de Investigaciones Biomédicas y Sanitarias (IUIBS), Universidad de Las Palmas de Gran Canaria, 35016 Las Palmas, Spain; 4 Clínica Veterinaria Escaleritas, Las Palmas de Gran Canaria, 35011 Las Palmas, Spain; 5 Departamento de Morfología, Universidad de Las Palmas de Gran Canaria, 35016 Las Palmas, Spain

**Keywords:** pigmentary keratitis, pug, tacrolimus, sirolimus, corneal pigmentation, tear film, ocular surface disease, brachycephalic breeds, topical immunomodulation

## Abstract

Pigmentary keratitis (PK) is a prevalent ocular surface disease in Pug dogs, yet comparative evidence on topical immunosuppressants remains limited. This prospective comparative clinical study evaluated the efficacy and safety of two agents with distinct mechanisms—tacrolimus, a calcineurin inhibitor, and sirolimus, an mTOR inhibitor—for the treatment of PK. Thirty-two Pugs (63 eyes) were randomly assigned to receive either 0.03% tacrolimus or 0.03% sirolimus three times daily for six months. Tear film quantity and quality were assessed using the Schirmer tear test, tear break-up time, and Ferning patterns, alongside serial clinical scoring of corneal pigmentation and ocular surface signs. Both treatments improved tear-film parameters, although only tacrolimus produced statistically significant increases in tear production and more frequent formation of a pigment-free “clear line,” indicating enhanced pigment regression. Pigment lightening and transparency recovery improved similarly in both groups. Adverse events—including blepharospasm, diffuse corneal oedema, and complicated ulcers—occurred more frequently in the sirolimus group, suggesting a comparatively less favorable short-term safety profile. Overall, both tacrolimus and sirolimus demonstrated therapeutic benefit in PK, although tacrolimus showed superior quantitative efficacy and better tolerability. Further long-term studies are warranted to clarify safety considerations and to optimize immunomodulatory strategies for this breed-specific condition. These findings suggest tacrolimus may be considered a first-line immunomodulatory therapy for PK in Pug dogs.

## Simple Summary

Pigmentary keratitis is a common eye condition in Pug dogs, in which dark pigment gradually spreads across the cornea, reducing comfort and eventually affecting vision. It is usually caused by chronic irritation, tear film instability, or the characteristic facial conformation of the breed. Because no fully effective treatment exists, managing this disease remains a challenge in veterinary practice. This study compared two topical immunomodulatory eye drops—tacrolimus and sirolimus—used to reduce inflammation and support tear production. Thirty-two Pugs received one of these treatments for six months, during which tear production, tear quality, and changes in corneal pigmentation were regularly assessed. Both treatments improved tear-film parameters, increased comfort, and reduced the amount of pigment on the corneal surface. However, tacrolimus produced clearer benefits, particularly in improving tear production and in creating a visible line of pigment regression. Some unwanted effects, including eye irritation and corneal ulcers, occurred more frequently in dogs treated with sirolimus. Overall, these findings suggest that tacrolimus may be the safer and more effective option for managing pigmentary keratitis in Pugs. This information may help veterinarians make better treatment decisions and improve the quality of life of affected dogs.

## 1. Introduction

Pigmentary keratitis (PK) is a chronic, progressive condition frequently observed in brachycephalic breeds, particularly in Pug dogs [1]. It is characterized by corneal melanosis secondary to chronic irritation, anatomical abnormalities, or tear film deficiencies [2]. Although various clinical approaches—including topical immunomodulators and surgical correction of palpebral anomalies—have been employed to manage PK, few studies have systematically evaluated the efficacy of these treatments in Pugs, despite the high breed-specific prevalence reported [3]. In Pugs, PK is considered a multifactorial disorder involving brachycephalic conformation, tear-film instability, nasal-fold friction, reduced corneal sensitivity, chronic microtrauma, and secondary immune-mediated inflammation. These factors contribute to chronic corneal melanosis, progressive fibrovascular tissue ingrowth and stromal opacity. Pugs represent a natural clinical model for severe, rapidly progressive corneal melanosis. Their extreme brachycephalic morphology, high global prevalence of PK and characteristic bilateral but asymmetric presentation make them ideal subjects for therapeutic studies evaluating immunomodulatory interventions.

Topical immunosuppressants such as cyclosporine and tacrolimus are commonly used in the management of PK and related ocular surface disorders [4,5,6,7,8,9,10]. While the exact mechanisms by which they reduce corneal pigmentation remain incompletely understood, these drugs are thought to reduce inflammation, inhibit neovascularization, and promote tear production, thereby contributing to improved corneal health and transparency. Although both tacrolimus and sirolimus are widely used systemic immunosuppressants, their comparative performance as topical ophthalmic agents has never been evaluated, despite their distinct mechanisms of action and their theoretical potential to influence corneal melanosis through different inflammatory and angiogenic pathways. In vitro, cyclosporine has been shown to inhibit melanogenesis and cell proliferation in human melanocytes [11,12], supporting a potential role in the management of corneal pigmentation.

Tacrolimus (formerly FK506) is a macrolide lactone derived from *Streptomyces tsukubaensis* with potent immunosuppressive properties [13]. It functions as a calcineurin inhibitor, blocking T-cell activation by preventing the nuclear translocation of the nuclear factor of activated T-cells (NFAT) and the subsequent transcription of interleukin-2 and other pro-inflammatory cytokines. Compared to cyclosporine, tacrolimus is reported to be 10–100 times more potent [14,15], and its efficacy in treating canine keratoconjunctivitis sicca (KCS) has been well documented [6,9,15,16]. In veterinary ophthalmology, topical tacrolimus is widely accepted as a therapeutic option, especially in patients unresponsive to cyclosporine [17,18,19].

Sirolimus (rapamycin) is another macrolide compound with immunosuppressive activity, produced by *Streptomyces hygroscopicus* [13,20]. Unlike tacrolimus, sirolimus binds to FK-binding protein 12 (FKBP12) and inhibits the mammalian target of rapamycin (mTOR), a serine/threonine kinase involved in cell growth, proliferation, and angiogenesis. By arresting the cell cycle at the G1/S transition and inhibiting T-cell proliferation, sirolimus represents a distinct and potentially advantageous pathway for immunomodulation. However, its inhibition of mTOR has also been associated with delayed epithelial healing and corneal oedema in experimental models [21,22], which could explain the adverse ocular effects occasionally observed in clinical settings.

While tacrolimus has become a standard treatment in canine ophthalmology, sirolimus remains relatively less explored. Preliminary studies in dogs have suggested that topical sirolimus may improve tear production in cases of refractory KCS, but response variability and the potential for local side effects necessitate further investigation [23,24,25]. The distinct mechanisms of action of these agents raise important questions regarding their comparative efficacy and safety in the management of pigmentary keratitis.

Although recent research has investigated the efficacy of subconjunctival liposomal sirolimus for the treatment of keratoconjunctivitis sicca (KCS) in dogs [26], no studies to date have directly compared topical sirolimus and tacrolimus in the management of pigmentary keratitis (PK), a distinct ocular surface disease primarily affecting brachycephalic breeds such as the Pug.

Despite the theoretical and experimental support for both compounds, a prospective, controlled evaluation of their comparative efficacy and safety in PK has not been previously conducted. The present study aimed to fill this gap by performing the first direct comparison between topical tacrolimus and sirolimus in Pug dogs with PK, assessing tear film function, clinical signs and objective indicators of corneal pigment regression.

## 2. Materials and Methods

### 2.1. Study Design and Ethical Approval

A prospective clinical study was conducted between 2014 and 2017 at the Veterinary Teaching Hospital, Faculty of Veterinary Medicine, University of Las Palmas de Gran Canaria (ULPGC, Canary Islands, Spain). The study adhered to national veterinary professional practice regulations and institutional standards of good clinical care. Because all animals were client-owned Pug dogs receiving standard topical ophthalmic treatments, the study was classified as a prospective clinical investigation rather than an experimental procedure. At the time of initiation (2014), the protocol was reviewed and authorised by the Ethical Committee for Clinical Veterinary Procedures of the Veterinary Teaching Hospital (ULPGC), which was the competent oversight body prior to the implementation of Real Decreto 53/2013. No experimental induction of disease or procedures exceeding routine ophthalmic examination and treatment were performed. Written informed consent was obtained from all owners before enrolment.

### 2.2. Data Collection and Animal Selection

Demographic variables including age, sex, fertility status and coat colour were recorded for all dogs examined. Coat colours were classified according to the Fédération Cynologique Internationale (FCI) standard (silver, apricot, fawn, black) and grouped into light (silver, apricot, fawn) and dark (black) categories to assess potential associations with pigmentary keratitis (PK). A detailed medical history was also obtained, including previous ocular diseases, prior medical or surgical treatments, and relevant systemic conditions.

A total of 110 Pug dogs (219 eyes), aged 1–13 years (mean ± SD: 6.58 ± 2.48 years), were examined clinically and ophthalmologically. Of these, 207 eyes (94.5%) exhibited signs of PK, whereas 12 eyes (5.5%) were considered clinically normal. This initial cohort constituted the epidemiological screening population and was not used for therapeutic comparisons.

Dogs eligible for the treatment phase had to meet the following criteria: Pug breed, age between 5 and 10 years, and presence of corneal pigmentation. Exclusion criteria included previous topical immunosuppressive treatment within the last six months, recent eyelid or corneal surgery, systemic or suspected systemic disease, and age outside the inclusion range. Eyes excluded after treatment initiation were removed from statistical analyses according to predefined criteria, and all excluded cases were documented to preserve transparency and avoid attrition bias.

Thirty-two Pug dogs (63 eyes) met the inclusion criteria and were randomly assigned to the two treatment groups. Baseline demographic characteristics were similar between groups. In the tacrolimus group, 22 eyes corresponded to dogs aged 4–7 years and 9 eyes to dogs aged ≥8 years; in the sirolimus group, 18 eyes corresponded to dogs aged 4–7 years and 14 eyes to dogs aged ≥8 years. Sex distribution was balanced (tacrolimus: 13 female, 18 male; sirolimus: 14 female, 18 male), as was fertility status (tacrolimus: 8 neutered, 23 intact; sirolimus: 10 neutered, 22 intact). These data confirm that both treatment groups were demographically comparable at baseline.

Group 1 (Tacrolimus) comprised 16 dogs (31 eyes) treated with 0.03% tacrolimus ophthalmic solution three times daily (TID) for six months. Group 2 (Sirolimus) comprised 16 dogs (32 eyes) receiving 0.03% sirolimus ophthalmic solution TID for the same duration. Randomization was performed using a computer-generated random number sequence. According to the standard clinical protocol of the Veterinary Teaching Hospital, dogs presenting mucopurulent ocular discharge and conjunctival inflammation at baseline received topical tobramycin (Tobrex™) for 7–10 days to control suspected secondary bacterial infection. After completion of antibiotic therapy, topical tacrolimus or sirolimus was initiated 24 h later, once no clinical signs suggestive of active infectious keratitis were present. No topical antibiotics were administered beyond this initial short course prior to immunomodulatory treatment.

No formal sample size calculation was performed owing to the exploratory nature of the study and the limited number of eligible Pug dogs presenting during the recruitment period.

### 2.3. Non-Treated Reference Population

During the initial epidemiological screening phase (110 Pugs; 219 eyes), 207 eyes showed pigmentary keratitis but did not enter the therapeutic trial. These eyes constituted the “non-treated group”. This group was not used as a control population; it served exclusively as a descriptive epidemiological reference, and no inferential or between-group statistical comparisons were performed.

### 2.4. Baseline Ocular Characteristics

Baseline ophthalmic findings recorded for all treated dogs included the presence of nasal fold contact, trichiasis, distichiasis, entropion, previous keratoconjunctivitis sicca, ocular discharge, corneal vascularization, inflammatory infiltrates, scleral pigmentation and any prior eyelid or corneal surgery. These variables are summarized in Table 1.

No statistically significant differences were identified between the tacrolimus and sirolimus groups for any baseline ocular variable, including nasal fold contact, trichiasis, distichiasis, entropion, previous keratoconjunctivitis sicca, ocular discharge, corneal vascularization, inflammatory infiltrates, or scleral pigmentation (all *p* > 0.10). These findings confirm that both groups were comparable at the start of the study and that no selection bias was present.

The severity of PK at baseline was graded using a four-tier scale ranging from Grade 1 (mild) to Grade 4 (severe), following the classification used in the original epidemiological study. The distribution of PK severity was similar between groups (Table 2). In the tacrolimus group, 2 eyes were classified as mild, 10 as moderate, 12 as moderate–severe, and 7 as severe. In the sirolimus group, 3 eyes were mild, 9 moderate, 12 moderate–severe, and 8 severe. No statistically significant differences in PK severity were observed between groups at baseline (all *p* > 0.10), confirming comparable disease status prior to treatment initiation.

### 2.5. Preparation of Topical Tacrolimus and Sirolimus

Both topical medications were compounded at the Pharmacy Service of the Veterinary Teaching Hospital (ULPGC). Tacrolimus 0.03% and sirolimus 0.03% ophthalmic solutions were prepared from pharmaceutical-grade powders (Fagron^®^, Rotterdam, The Netherlands) using an identical sterile aqueous vehicle containing polysorbate-80 and 0.3% hypromellose as a viscosity-enhancing agent. All formulations were prepared under laminar-flow sterile conditions, filtered at 0.22 μm, dispensed in opaque single-use bottles, and used within 30 days of preparation. No preservatives were added to avoid epithelial irritation. Using the same vehicle for both formulations ensured that any observed clinical differences were attributable to the active compounds rather than excipient variability.

### 2.6. Owner Consent and Compliance

Owners received comprehensive written instructions regarding treatment administration and follow-up visits. Written informed consent was obtained, including confirmation of their ability to administer eye drops TID for six months and attend scheduled check-ups every two months.

### 2.7. Clinical Evaluation and Follow-Up

All ophthalmic examinations were performed using a portable slit-lamp biomicroscope (Kowa SL-15^®^, Tokyo, Japan), a direct ophthalmoscope (Heine Beta 200^®^, Gilching, Germany) and fluorescein sodium strips (Fluostrip^®^, Biovision, Milpitas, CA, USA). Tear break-up time was recorded under cobalt-blue illumination using a digital chronometer. Central, nasal and temporal corneal thickness were measured using an ultrasonic pachymeter (Accutome PachPen^®^, Malvern, PA, USA). Three consecutive measurements were taken at each location, and the mean value was used for analysis. Standardized digital photographs were obtained using a Canon EOS 700D macro lens system and under identical lighting conditions, fixed camera-to-eye distance and constant macro-lens settings to ensure reproducibility.

Tear Film Break-Up Time (TBUT) was measured after instilling a fluorescein sodium strip moistened with sterile saline into the lower fornix. The time from the last blink to the first dark spot on the tear film was assessed under cobalt-blue illumination. Three measurements were obtained per eye, and the mean value was used for analysis.

The Tear Ferning Test was performed following the methodology of Rolando [27]. A 1 μL sample of tear fluid was collected from the inferior tear meniscus using a sterile microcapillary tube, placed on a glass slide, and allowed to air-dry at room temperature (22–24 °C) and 40–50% humidity. Ferning patterns were classified into four types: Type I (uniform dense ferns, normal), Type II (partially disrupted ferns), Type III (fragmented or irregular ferns), and Type IV (absent or amorphous crystallization, poor tear quality).

Clinical evaluations were conducted every two months over a six-month period. Tear film status was assessed using the Schirmer tear test (STT) for quantitative analysis, and tear break-up time (TBUT) and tear Ferning test (TFT) for qualitative analysis. The fluorescein test was used to assess corneal surface integrity [28,29]. Serial photographic documentation and repeated ultrasonic pachymetry (nasal and temporal zones) were performed to monitor corneal pigmentation changes.

The percentage of the corneal surface affected by pigmentation was estimated from standardized photographs using a schematic corneal grid divided into quadrants. Pigmentation extent was recorded as the proportion of the corneal surface (%) exhibiting visible pigment.

Clinical signs evaluated included blepharospasm, conjunctival hyperemia, pruritus, corneal oedema, neovascularization, inflammatory infiltrates, and scleral pigmentation. Each parameter was scored using a 5-point scale (1: absent; 5: severe). Ocular discharge was categorized as absent, serous, mucous, or purulent.

**Figure 1:**
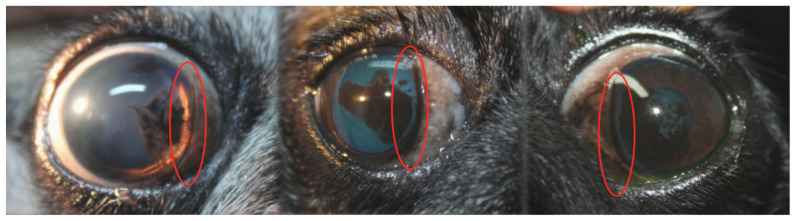
Representative slit-lamp photographs from different Pug dogs illustrating the formation of a clear line in pigmentary keratitis. The clear line corresponds to a pigment-free demarcation zone between the physiological limbal pigmentation and the corneal pigmentation associated with pigmentary keratitis, indicating a peripheral-to-central pattern of pigment regression. Areas of clear line formation are highlighted (red ovals).

Three indicators were used to assess corneal pigment regression throughout the study, based on standardized digital photographs evaluated independently by two masked observers, both of whom were fully blinded to treatment allocation.

(A) Clear line: defined as a visible, pigment-free demarcation between physiological limbal and the central corneal pigmentation associated with pigmentary keratitis, reflecting a peripheral-to-central pattern of pigment regression. This parameter was graded on a four-point ordinal scale from 1 (absent) to 4 (marked, involving ≥50% of the limbal circumference), based on standardized photographic documentation. Representative examples of clear line formation over time are shown in Figure 1.

(B) Pigment lightening: representing increased translucency of previously dense pigment, scored from 1 (none) to 4 (heavy, with >50% of the pigmented area showing reduction in opacity).
(C) Transparency recovery: assessing overall restoration of corneal clarity at the end of the six-month treatment period, scored from 1 (none) to 4 (intense, with visibility of iris details).

Pigment density was assessed based on the degree of stromal opacity and the visibility of underlying iris structures, following a standardized photographic reference scale derived from the baseline epidemiological study. All clinical scores used in this study were based on predefined internal grading criteria developed during the preliminary epidemiological phase and assessed by two masked observers. Inter-observer discrepancies were resolved by consensus.

For the purposes of this study, “inflammatory infiltrates” referred to focal or diffuse whitish anterior stromal opacities without epithelial ulceration, hypopyon, keratomalacia or stromal melting. These lesions were interpreted as sterile immune-mediated keratitis rather than infectious keratitis, consistent with the absence of ulceration, stromal loss, purulent discharge or hypopyon.

### 2.8. Statistical Analysis

All statistical analyses were conducted using IBM SPSS Statistics 23. A *p*-value < 0.05 was considered statistically significant. Descriptive statistics included means, standard deviations, and frequency distributions. For group comparisons, ordinal variables were analysed using the Mann–Whitney U test, and categorical variables using chi-square or Fisher’s exact test as appropriate. Analysis of variance (ANOVA) was applied to compare quantitative data between groups, with Levene’s test used to assess homogeneity of variance. Where appropriate, non-parametric tests were used: Mann–Whitney U or Kruskal–Wallis for independent samples, and Friedman or Wilcoxon tests for repeated measures. Odds ratios (OR) were calculated for factors associated with severe PK. Efficacy analyses were conducted per protocol and included only eyes that completed the six-month follow-up. Safety analyses included all eyes exposed to at least one dose of treatment (intention-to-treat).

## 3. Results

Of the 31 eyes initially enrolled in the tacrolimus group, six eyes (three animals) were excluded at the first follow-up due to incorrect administration of ophthalmic drops, and one eye was excluded following the unrelated death of the animal. As a result, 24 eyes completed the study in this group. In the sirolimus group, 10 eyes were excluded: six due to complicated corneal ulcers (three animals), one due to a foreign body-induced ulcer, two due to moderate bilateral diffuse corneal oedema (one animal), and one animal (two eyes) died for unrelated reasons. These excluded cases were not included in efficacy analyses but were documented for safety evaluation. Ultimately, 22 eyes completed the study in the sirolimus group. As adverse events were observed in several cases, the number of eyes (n) varied across parameters. Our findings are summarized in Table 3.

### 3.1. Tear Film Quantity—Schirmer Tear Test (STT)

Both treatment groups demonstrated improved tear production over the six-month period. In the tacrolimus group, STT values increased significantly from 15.35 ± 5.33 mm/min at baseline to 20.88 ± 4.51 mm/min at six months. In the sirolimus group, values rose from 14.47 ± 6.19 mm/min to 15.91 ± 5.45 mm/min, although this change was not statistically significant. Wilcoxon signed-rank tests revealed a significantly greater improvement in the tacrolimus group at months 2 (*p* = 0.011), 4 (*p* = 0.031), and 6 (*p* = 0.002) (Table 3). These baseline values are consistent with those reported in the epidemiological study of Pugs with PK performed at the same institution, in which the mean STT was 16.26 ± 5.78 mm/min (N = 219).

### 3.2. Tear Film Quality—Ferning Test

Both groups showed a shift toward more favorable Ferning patterns (Types I and II). Inter-group comparisons revealed significant differences at baseline (*p* = 0.005) and at month 2 (*p* = 0.025), with a more favorable distribution in the tacrolimus group. However, no sustained intra-group improvement in Ferning patterns was observed beyond month 2 in either group, and no further significant inter-group differences were detected at subsequent time points. Overall, changes were less pronounced in the sirolimus group.

### 3.3. Tear Film Stability—Tear Break-Up Time (BUT)

Tear break-up time increased in both treatment groups, but without statistically significant inter-group differences. In the tacrolimus group, BUT improved from 4.94 ± 2.19 s to 6.38 ± 1.71 s, and in the sirolimus group from 5.88 ± 2.45 s to 6.32 ± 2.17 s. Intra-group analyses showed significant improvement at month 2 for both treatments (tacrolimus: *p* = 0.010; sirolimus: *p* = 0.028). A trend toward longer BUT values in the tacrolimus group was observed at month 4 (*p* = 0.061), although this difference did not reach statistical significance.

### 3.4. Corneal Thickness—Ultrasonic Pachymetry

Corneal thickness did not differ significantly between groups or time points. Medial values in the tacrolimus group ranged from 776.53 ± 160.69 µm to 757.73 ± 106.15 µm, and lateral values from 680.90 ± 147.19 µm to 674.14 ± 108.55 µm. In the sirolimus group, medial values fluctuated between 775.94 ± 135.85 µm and 785.45 ± 199.85 µm, and lateral values between 712.44 ± 140.92 µm and 730.32 ± 154.03 µm. No significant changes were detected (all *p* > 0.1). Pachymetry did not correlate with pigment clearance and showed high regional variability, limiting its usefulness as an outcome measure.

### 3.5. Adverse Ocular Events

Six corneal ulcers were detected throughout the study: all simple ulcers occurred in the tacrolimus group, whereas the sirolimus group presented a higher incidence of complicated ulcers, one of which was related to a foreign body. Blepharospasm was absent at baseline but appeared in six eyes of the sirolimus group at month 2, four of which were associated with the presence of corneal ulceration. Though more frequent in this group, the difference was not statistically significant (*p* = 0.073). At month 4, mild blepharospasm was recorded in one animal (excluded from the study due to oedema), and no cases were observed at month 6.

### 3.6. Hyperemia and Ocular Pruritus

Hyperemia was present at baseline in both groups but decreased significantly over time (tacrolimus: *p* < 0.001; sirolimus: *p* = 0.046). Although inter-group differences were observed at month 2, they were not statistically significant at months 4 and 6. Ocular pruritus, initially mild, resolved completely in the tacrolimus group, whereas a transient increase was noted at months 2 and 4 in the sirolimus group, with no significant inter-group differences at the study endpoint.

### 3.7. Corneal Oedema and Inflammatory Infiltrate

While some cases of mild to moderate oedema were noted at baseline, oedema increased in the sirolimus group at months 2 and 4. A statistically significant difference was observed between groups at month 4 (*p* = 0.019), with the sirolimus group showing a higher incidence. Additionally, both groups exhibited significant differences compared to baseline, especially the sirolimus group (*p* = 0.001). Inflammatory infiltrates were generally absent, although the sirolimus group showed a significant increase over time (*p* = 0.019).

### 3.8. Vascularization

Corneal vascularization increased in both groups, coinciding with pigment lightening or removal. A significant increase in vessel density was observed at month 4 in the sirolimus group (*p* = 0.018) and at month 6 in both groups. However, no statistically significant inter-group differences were detected. Increased vessel visibility was interpreted as exposure of pre-existing vessels following pigment regression rather than new pathological neovascularization.

### 3.9. Scleral Pigmentation

Significant reductions in scleral pigmentation were observed in both groups. Inter-group comparisons revealed that sirolimus was significantly more effective at months 4 and 6 (*p* = 0.028 and *p* = 0.037, respectively), suggesting a differential efficacy in this specific parameter.

### 3.10. Ocular Discharge

Both treatments resulted in significant improvement in ocular discharge (*p* < 0.001 at all time points). By month 2, most eyes showed no discharge, while at months 4 and 6, serous discharge predominated. No significant differences were found between treatment groups.

### 3.11. Clear Line Formation

The appearance of a clear line—indicative of pigment regression—was significantly more frequent in the tacrolimus group, with statistical differences at months 2 (*p* = 0.029) and 4 (*p* = 0.039). This feature was also significantly improved over time in both groups individually. The clear line reflects a peripheral-to-central regression pattern characteristic of immunomodulatory response rather than epithelial remodeling.

### 3.12. Pigment Lightening and Transparency Recovery

Both treatments led to significant pigment lightening (tacrolimus: *p* < 0.001; sirolimus: *p* = 0.017), although inter-group differences were not significant. Transparency recovery at six months was comparable in both groups, with no statistically significant difference observed.

**Figure 2:**
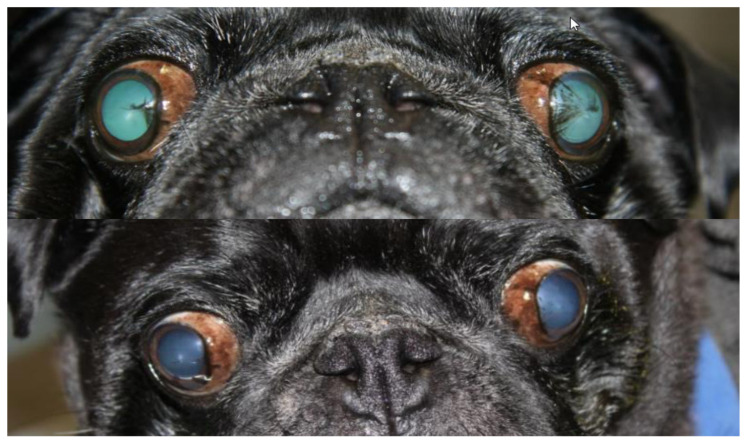
Slit-lamp photographs of a Pug dog with pigmentary keratitis treated with topical tacrolimus. The (**upper**) image shows the eye at baseline, with extensive corneal pigmentation prior to treatment. The (**lower**) image shows the same eye after six months of treatment, demonstrating marked pigment lightening and recovery of corneal transparency.

**Figure 3:**
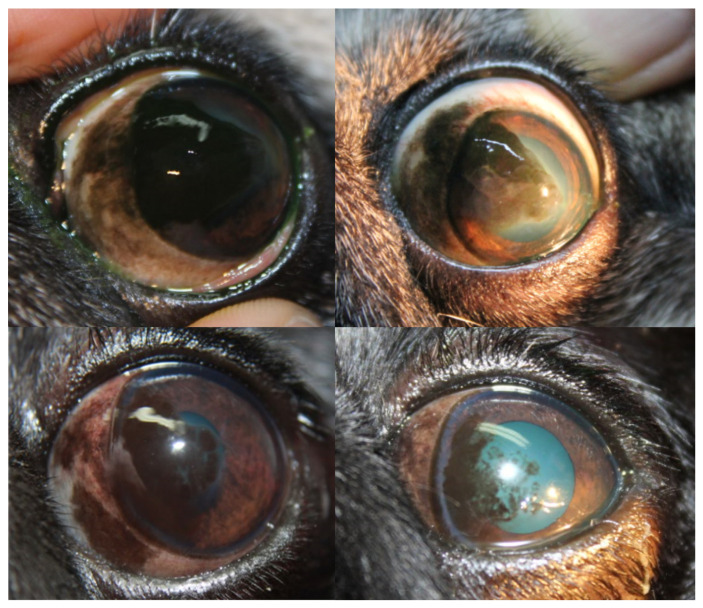
Slit-lamp photographs from two additional representative Pug dogs with pigmentary keratitis illustrating clinical improvement following treatment. In each case, baseline images (**left**) show marked corneal pigmentation, whereas post-treatment images (**right**) demonstrate pigment lightening and partial recovery of corneal transparency.

Representative slit-lamp photographs illustrating corneal pigmentation changes are shown in Figure 2 and Figure 3. Figure 2 presents a representative case treated with topical tacrolimus, with baseline appearance shown in the upper image and post-treatment appearance after six months shown in the lower image, demonstrating marked pigment lightening and recovery of corneal transparency. Figure 3 includes before-and-after images from two additional dogs, further illustrating the clinical improvement observed following treatment.

## 4. Discussion

This study presents a novel comparison between two topical immunosuppressants with distinct mechanisms of action—tacrolimus, a calcineurin inhibitor, and sirolimus, an mTOR inhibitor—in the treatment of pigmentary keratitis (PK) in Pug dogs. To ensure the comparability of results, both drugs were formulated using identical excipients and concentrations (0.03%) and were administered under the same protocol (TID for six months). To our knowledge, this is the first prospective study directly comparing these two agents in this clinical context. Given the breed-specific nature and progressive course of PK in Pugs, head-to-head comparisons of immunomodulators are essential to guide evidence-based therapeutic decision-making.

In accordance with the standard clinical protocol of the Veterinary Teaching Hospital, eyes presenting conjunctival inflammation and mucopurulent discharge received topical tobramycin for 7–10 days prior to immunomodulatory therapy. Tacrolimus or sirolimus was started 24 h after completing antibiotic treatment, once no clinical signs suggestive of active infectious keratitis were present. No ulceration, stromal melting or purulent discharge was detected in any case. Screening for active infectious keratitis prior to initiating topical calcineurin or mTOR inhibitors is recommended in chronic keratitis management to avoid exacerbation of the underlying bacterial disease.

Previous studies comparing immunosuppressants for canine keratoconjunctivitis sicca (KCS) have largely focused on calcineurin inhibitors [30,31]. Ofri et al. [32] compared pimecrolimus and cyclosporine, while Hendrix et al. [15] evaluated tacrolimus versus cyclosporine, albeit under heterogeneous conditions including continued use of prior medications. Radziejewski and Balicki [31] also assessed tacrolimus versus cyclosporine, but again without standardization of vehicle or frequency. By contrast, our study aimed to isolate the effects of the active ingredients by controlling these variables. It is important to note that none of the previous studies evaluated immunomodulatory therapy in pigmentary keratitis, a disease with a markedly different pathophysiology from that of KCS. Therefore, direct extrapolation of their findings to PK is limited.

Owner compliance remains an unavoidable variable in clinical trials involving companion animals. Nevertheless, both tacrolimus and sirolimus have previously demonstrated good tolerability. Fonzar et al. [33] confirmed the ocular safety of subconjunctival and intravitreal sirolimus in horses, while Lance et al. [34] reported sustained ocular compatibility of sirolimus-loaded implants in rabbits. Tacrolimus has also shown a favorable safety profile in both veterinary [15,16] and human studies [11,35]. Nevertheless, the FDA has issued warnings regarding the long-term dermal use of tacrolimus in humans due to a potential risk of carcinogenesis, though its relevance to ocular use in dogs remains speculative [15].

### 4.1. Tear Film Quantity and Quality

In line with previous reports [15,16], our findings confirmed the efficacy of tacrolimus in increasing tear production, with statistically significant improvements at all measured time points. Sirolimus also led to increased Schirmer values, though to a lesser extent and without statistical significance. Linares-Alba et al. [36] reported marked STT improvements following subconjunctival sirolimus in refractory KCS cases, suggesting a potential role in tear stimulation. This greater magnitude of improvement observed with tacrolimus may reflect its more potent inhibition of T-cell–mediated lacrimal gland inflammation compared with mTOR inhibition.

Tear film quality, assessed by tear break-up time (BUT) and the Ferning test (TFT), showed early stabilization in both groups, with significant inter-group differences favoring tacrolimus at month 2, but without evidence of sustained intra-group improvement over time. BUT values increased significantly at two months in both treatment groups, consistent with the findings of Linares-Alba et al. [36]. While no intra-group differences were detected in TFT scores, inter-group comparisons showed significantly better tear quality in the tacrolimus group at month 2. The lack of significant intra-group improvement in TFT scores beyond month 2 suggests early stabilization of tear-film microstructure rather than progressive enhancement.

### 4.2. Corneal Thickness and Clinical Relevance

Ultrasonic pachymetry was explored as a marker of disease severity and response to treatment. Although no significant changes in thickness were observed, we hypothesize that pachymetric values may reflect the extent and chronicity of pigment accumulation and may thus aid in prognostic evaluation or surgical planning. Alario and Pirie [37] similarly advocated for its use in corneal surgery for PK. Although pachymetry was not sensitive to treatment-induced changes, its inclusion remains clinically relevant because corneal thickness may influence diagnostic interpretation and surgical decision-making in chronic PK.

### 4.3. Adverse Effects and Safety Considerations

Corneal ulceration, blepharospasm and diffuse corneal oedema were more frequent in the sirolimus group. While causality remains uncertain—given the breed’s predisposition to ulceration and the presence of foreign bodies in some cases—these findings warrant caution. All corneal ulcers in the tacrolimus group were simple, whereas those in the sirolimus group were complicated and often bilateral. This difference, while not statistically significant, is clinically relevant. Given the absence of ulceration at baseline, the development of complicated ulcers in the sirolimus group raises the possibility—but does not prove—a treatment-related susceptibility.

Hyperemia resolved significantly in both groups, though more consistently in the tacrolimus group. Ocular pruritus, initially present in both groups, disappeared entirely in the tacrolimus group and persisted mildly in the sirolimus group, potentially due to associated ulcerations. Diffuse oedema increased significantly in both groups, but more so in the sirolimus group (*p* = 0.019), suggesting a possible adverse effect.

Corneal vascularization increased over time in both groups, likely secondary to pigment regression and epithelial remodeling. However, this contrasts with prior studies in KCS, where both sirolimus and tacrolimus reduced vascularisation [31,36]. The difference may be attributed to the distinct pathophysiology of PK compared with that of KCS. Importantly, the apparent increase in vascularization must be interpreted cautiously. In several eyes, regressing stromal pigment revealed vascular profiles that had previously been obscured, creating the impression of new vessel formation despite no true angiogenic progression [3]. As proposed in prior work on chronic keratitides and supported by observations in the present population, stromal pigment can mask pre-existing superficial or mid-stromal vessels, which become more visible as pigmentation diminishes [38,39]. In our cohort, these vascular changes were not accompanied by signs of active inflammation, supporting the interpretation that the increased visibility of vessels reflected pigment regression rather than disease progression. Therefore, increases in visible vascular profiles should not be interpreted as worsening keratitis unless accompanied by concurrent inflammatory signs.

The inflammatory infiltrates detected during follow-up were non-ulcerative and lacked clinical indicators of infection (no purulent discharge, stromal melting, or hypopyon). Their behavior was consistent with sterile immune-mediated keratitis rather than infectious disease. Inflammatory infiltrates remained generally absent, though a significant increase was noted in the sirolimus group. This may relate to a higher proportion of severe PK cases in this group. Similarly, scleral pigmentation decreased significantly in both groups, with sirolimus demonstrating superior efficacy at 4 and 6 months.

Corneal oedema was mild and transient in the majority of cases, predominantly peripheral, and not associated with endothelial decompensation or epithelial ulceration. These episodes likely reflected focal epithelial stress or transient stromal hydration changes rather than drug toxicity. The higher frequency in the sirolimus group may relate to variability in corneal penetration or differences in ocular surface tolerability compared with tacrolimus. mTOR inhibition has been shown to transiently alter epithelial tight-junction integrity, which may contribute to these episodic oedematous changes.

To better understand these safety observations, it is worth considering the pharmacodynamic profiles of each agent. The difference in adverse event profiles observed between tacrolimus and sirolimus may be partly explained by their distinct mechanisms of action. While tacrolimus inhibits calcineurin and thus suppresses T-cell activation and pro-inflammatory cytokine release, sirolimus exerts its effect via mTOR inhibition, a pathway involved in cell proliferation, angiogenesis, and epithelial regeneration [6,13,16,18,33]. mTOR suppression has been associated with delayed epithelial wound healing and increased vascular permeability in corneal models, potentially accounting for the higher incidence of corneal oedema and complicated ulcers observed in the sirolimus group [21,22]. These findings suggest that while both drugs are immunosuppressive, their downstream cellular effects may differentially impact ocular surface repair and homeostasis.

### 4.4. Indicators of Clinical Efficacy

In addition to standard clinical signs, this study assessed novel parameters such as the “clear line” and transparency recovery to characterize treatment response more precisely. The “clear line,” representing a demarcation between limbal and corneal pigmentation, was more frequently observed in the tacrolimus group, with significant differences at months 2 and 4. This parameter may serve as an early, sensitive indicator of therapeutic response, particularly in diseases where pigment regression precedes structural corneal changes. Both groups showed significant pigment lightening over time, without inter-group differences. Transparency recovery was comparable between groups at six months. However, the degree of transparency restoration was limited by the chronicity of stromal melanosis, consistent with the well-recognized difficulty of reversing advanced PK.

Our results support those of Linares-Alba et al. [36], who reported significant improvement in ocular appearance with sirolimus. By contrast, Hendrix et al. [15] found no significant improvement in clinical signs when comparing tacrolimus and cyclosporine, possibly due to their shorter follow-up. Ofri et al. [32] reported greater clinical improvement with pimecrolimus than with cyclosporine.

Unlike keratoconjunctivitis sicca, which primarily affects tear production, pigmentary keratitis involves progressive corneal melanosis secondary to chronic ocular surface irritation and tear film instability. Therefore, its pathophysiology and clinical response to immunomodulatory treatment may differ substantially from those observed in KCS [26], highlighting the need for disease-specific therapeutic investigations such as the present study.

Although tacrolimus has been extensively studied in veterinary ophthalmology, sirolimus remains a relatively novel agent in this context. A preliminary study by Spatola et al. [23] explored the effects of topical sirolimus 0.02% in both healthy dogs and dogs with refractory KCS, showing increased tear production in a subset of treated eyes. However, the therapeutic response was inconsistent, and some dogs showed minimal benefit, underscoring the need for further evaluation. Our results add to this emerging evidence by suggesting that while sirolimus may offer immunomodulatory benefits, its safety profile warrants caution. Future studies could explore alternative concentrations, formulations, or administration protocols to determine its optimal role and safety profile in canine ophthalmology.

### 4.5. Limitations and Future Directions

This study had limitations, including a relatively small sample size and absence of long-term follow-up. Although both drugs were generally well tolerated, the therapeutic window for topical immunosuppressants in dogs remains undefined, and long-term safety remains uncertain. The potential association between chronic immunosuppression and ocular neoplasia, such as squamous cell carcinoma, remains to be clarified [40,41].

The absence of SPOTS scoring [42] should be interpreted within the historical context of the study design, preceding the scale’s development, rather than as a methodological limitation of the current work. Ocular tolerability was instead assessed through objective clinical parameters (oedema, vascularization, infiltrates, discharge, ulceration), all of which were documented photographically and reviewed by masked observers. Future prospective studies may benefit from incorporating SPOTS scoring to facilitate cross-study comparisons.

These findings underscore the need for larger, multicentre trials and for standardized outcome metrics tailored specifically to pigmentary keratitis.

## 5. Conclusions

This study demonstrates that both topical tacrolimus and sirolimus improved tear film parameters, although tacrolimus produced statistically significant increases in tear production. Quantitatively, tacrolimus showed superior efficacy in stimulating tear production over the six-month treatment period. Qualitatively, both treatments led to improved tear film stability, with tacrolimus showing a greater early normalizing effect, most evident at month 2.

Although both treatments contributed to pigment lightening and recovery of corneal transparency, tacrolimus was more effective in achieving the formation of a “clear line,” a potential indicator of treatment success. In contrast, sirolimus demonstrated greater efficacy in reducing scleral pigmentation and mid-term corneal oedema, whereas pruritus resolved more consistently with tacrolimus.

While the incidence of corneal ulceration and blepharospasm was higher in the sirolimus group, it remains unclear whether these findings were directly related to the drug or influenced by the breed’s predisposition. Both treatments were associated with increased visibility of corneal vessels due to pigment regression. Inflammatory infiltrates were uncommon but more frequently observed with sirolimus.

In summary, tacrolimus appears to be more effective for improving tear production and corneal clarity, while sirolimus may offer additional benefits in managing scleral pigmentation and selected ocular surface inflammatory changes. Both drugs represent promising treatment options for PK in brachycephalic breeds, although sirolimus may require cautious use pending further long-term safety evaluation.

## Figures and Tables

**Table 1:** Baseline Ocular Characteristics of Treated Pug Dogs with Pigmentary Keratitis.

Ocular Variable	Tacrolimus (n = 31 Eyes)	Sirolimus (n = 32 Eyes)
Nasal fold contact	29/31 (93.5%)	30/32 (93.7%)
Trichiasis	28/31 (90.3%)	29/32 (90.6%)
Distichiasis	4/31 (12.9%)	5/32 (15.6%)
Entropion (medial/total)	7/31 (22.6%)	6/32 (18.7%)
History of KCS	3/31 (9.7%)	4/32 (12.5%)
Ocular discharge at baseline	21/31 (67.7%) (serous: 10; mucous: 6; purulent: 5)	21/32 (65.6%) (serous: 14; mucous: 4; purulent: 3)
Corneal vascularization	18/31 (58.1%)	19/32 (59.3%)
Inflammatory infiltrates	2/31 (6.4%)	3/32 (9.3%)
Scleral pigmentation	16/31 (51.6%)	18/32 (56.2%)
Prior eyelid surgery	0/31 (0%)	1/32 (3.1%)
Prior corneal surgery	0/31 (0%)	0/32 (0%)

**Table 2:** Baseline Severity of Pigmentary Keratitis in Treated Eyes.

PK Severity Grade	Tacrolimus (n = 31 Eyes)	Sirolimus (n = 32 Eyes)
Grade 1—Mild	2 (6.5%)	3 (9.4%)
Grade 2—Moderate	10 (32.3%)	9 (28.1%)
Grade 3—Moderate–Severe	12 (38.7%)	12 (37.5%)
Grade 4—Severe	7 (22.6%)	8 (25.0%)

**Table 3:** Tear film parameters and corneal thickness in Pug dogs with pigmentary keratitis treated with topical tacrolimus or sirolimus, and in a non-treated reference population.

	Group	Initial (N = 31)		Month 2 (N = 25)		Month 4 (N = 24)		Month 6 (N = 24)		No Treatment Group	
**Schirmer test (mm/min)**	**Tacrolimus**	15.35 ± 5.33		20.52 ± 4.92		20.54 ± 6.76		20.88 ± 4.51		16.26 ± 5.78 (N = 219)	
	**Sirolimus**	14.47 ± 6.19		16.44 ± 5.88		17.04 ± 5.91		15.91 ± 5.45			
	*p*-value	*p* = 0.546		***p* = 0.011**		***p* = 0.031**		***p* = 0.002**			
**Ferning Test** **(N in type I, II, III, IV)**	**Tacrolimus**	6, 16, 3, 6		3, 14, 3, 5		1, 16, 4, 3		2, 8, 7, 7		21,65,51,34 (N = 171)	
	**Sirolimus**	1, 9, 14, 8		1, 8, 13, 3		2, 9, 8, 5		3, 7, 8, 4			
	*p*-value	*p* = 0.005		***p* = 0.025**		*p* = 0.248		*p* = 0.785			
**BUT** **(seconds)**	**Tacrolimus**	4.94 ± 2.19		6.80 ± 1.73		7.46 ± 2.55		6.38 ± 1.71		5.90 ± 2.74 (N = 207 *)	
	**Sirolimus**	5.88 ± 2.45		6.96 ± 2.15		6.21 ± 1.91		6.32 ± 2.17			
	*p*-value	*p* = 0.114		*p* = 0.773		*p* = 0.061		*p* = 0.922			
**Ultrasonic pachymetry** **(µm)**		Med	Lat	Med	Lat	Med	Lat	Med	Lat	Med	Lat
	**Tacrolimus**	776.53 ± 160.69	680.90 ± 147.19	754.41 ± 126.53	694.77 ± 141.10	762.45 ± 148.25	706.45 ± 131.73	757.73 ± 106.15	674.14 ± 108.55	769.97 ± 143.59 (N = 154)	702.75 ± 135.70 (N = 154)
	**Sirolimus**	775.94 ± 135.85	712.44 ± 140.92	765.15 ± 197.86	713.58 ± 126.21	834.83 ± 165.26	727.58 ± 127.64	785.45 ± 199.85	730.32 ± 154.03		
	*p*-value	0.987	0.392	0.827	0.628	0.126	0.584	0.569	0.169		

* The number of eyes varies due to the availability of complete tear-film recordings. The non-treated group was included as an epidemiological reference only and was not used for inferential statistical comparisons.

## Data Availability

The original contributions presented in this study are included in the article. Further inquiries can be directed to the corresponding author(s).
